# Changed Trends in Utilization and Substitution Pattern of Non-National Immunization Program Vaccines in Central China, 2011–2024

**DOI:** 10.3390/vaccines14010016

**Published:** 2025-12-23

**Authors:** Lei Wang, Hao Li, Ling Zhang, Dan Li

**Affiliations:** 1 School of Public Health, Wuhan University of Science and Technology, Wuhan 430065, China13407199801@163.com (H.L.);; 2 Institute of Infectious Disease Prevention and Control, Hubei Provincial Center for Disease Control and Prevention, Wuhan 430079, China; 3 School of Economics and Management, China University of Geosciences, Wuhan 430074, China; 4 Da Ye City Center for Disease Control and Prevention, Huangshi 435100, China

**Keywords:** non-National Immunization Program vaccine, National Immunization Program vaccine, changed trend, substitution rate, immunization strategy

## Abstract

**Objective:** To explore the problems with non-National Immunization Program vaccinations in Hubei Province and to provide the basis for follow-up vaccination and management. **Methods:** Vaccination data on non-NIP/NIP vaccine doses were extracted from the Hubei Provincial Immunization Planning Information Management System. Descriptive epidemiological analyses were conducted to examine dose administration, vaccine-type composition, regional distribution, and substitution patterns. The trend χ^2^ test was used to assess temporal significance. Multistage regression analysis was performed using Joinpoint software. **Results:** From 2011 to 2024, a total of 91,009,259 doses (annual average: 6,500,661) with 35 types of non-NIP vaccines were administered in Hubei Province, China. The top five vaccines by doses administered were influenza vaccine, rabies vaccine, Hemophilus influenzae type b conjugate vaccine, varicella attenuated live vaccine, and enterovirus 71 inactivated vaccine. Before 2024 (2011–2023), vaccine utilization showed a long-term upward trend: per 10,000, population usage rose from 657.07 (2011) to a peak of 2393.21 (2023) (Increase: 264.22%, χ^2^ = 138.62, *p* < 0.05) (AAPC = 10.92%, *p* < 0.05) and non-NIP’s share of total vaccines increased from 25.52% (2011) to 65.95% (2023), (Increase: 154.33%, χ^2^ = 89.47, *p* < 0.05) (AAPC = 8.74%, *p* < 0.05). A notable reversal occurred in 2024. Non-NIP doses dropped from 13,971,544 (2023) to 10,238,861 (2024) with population usage falling from 2393.21 (2023) to 1755.03 (2024) (decrease: 26.66%) per 10,000, with the top three declines being in inactivated polio vaccine (IPV) (decrease: 49.53%), influenza vaccine (decrease: 44.21%), and oral rotavirus attenuated live vaccine (decrease: 43.50%). The total number of substitutive non-National Immunization Program (non-NIP) vaccine doses administered reached 16,618,755, with an overall substitution rate of 10.10%. This rate showed a steady upward trend from 5.57% in 2011 to 24.74% in 2023 (trend χ^2^ = 15.11, *p* < 0.05), yet it increased to 28.03% in 2024. **Conclusions:** Non-NIP vaccines and NIP-substitute use grew steadily for over a decade, then contracted sharply in 2024. Decision-makers should investigate the sudden dip, differentiate discretionary from replacement demand, and reallocate funds to sustain equity and prevent further erosion of coverage.

## 1. Introduction

Vaccination is one of the most effective measures for preventing infectious diseases. In China, a two-tier immunization system is operated: the National Immunization Program (NIP) provides free, compulsory vaccines, whereas non-NIP vaccines are voluntary and paid for by the patient. The non-NIP vaccines are vaccines other than immunization program vaccines, which are classified according to the annual national immunization catalog. During the past decade, rising disposable income, recurrent disease outbreaks, and industry expansion have fueled unprecedented demand for non-NIP products [1,2]. Yet, national surveillance focuses almost exclusively on NIP coverage, creating a large evidence gap about the real-world scale and composition, and the substitution effect of market-driven vaccines. Understanding these dynamics is essential for calibrating procurement budgets, maintaining equity, and updating immunization policy [3,4,5].

Hubei Province, located in central China, with 58 million residents and a fully digitized Immunization Information Management System, provides an ideal natural laboratory [6]. Previous single-year or hospital-based studies have reported a high willingness-to-pay for non-NIP vaccines, but none have examined the longitudinal trends, geographical heterogeneity, or replacement of NIP antigens by using population-level data [7]. We therefore conducted a comprehensive 14-year analysis (2011–2024) to quantify non-NIP vaccine utilization, vaccine types, substitution patterns, age strata, and regional distribution in Hubei Province.

This study presents a comprehensive longitudinal analysis of non-National Immunization Program (non-NIP) vaccine utilization and substitution trends in Hubei Province, central China, from 2011 to 2024. Our findings reveal a decade-long upward trajectory in non-NIP vaccine use, followed by a notable decline in 2024. This trend reflects evolving public health priorities, vaccine policy changes, and shifting public perceptions in recent years in central China.

## 2. Materials and Methods

### 2.1. Data Sources

Data on the number of doses administered for vaccines under the National Immunization Program (NIP) and for non-NIP vaccines were extracted from the Hubei Provincial Immunization Planning Information Management System. Annual population figures for 2011–2024 were obtained from the Hubei Statistical Yearbook.

### 2.2. Non-NIP Vaccine Categories

Between 2011 and 2024, a total of 35 non-NIP vaccines were in use in Hubei Province. The annual classification criteria for non-NIP vaccines are updated year by year. These include the hepatitis B vaccine (HepB), Hemophilus influenzae type b conjugate vaccine (Hib), varicella attenuated live vaccine (VarV), influenza vaccine (InfV), quadrivalent influenza vaccine (InfV-4), 23-valent pneumococcal polysaccharide vaccine (PPV23), rabies vaccine (RabV), DTaP-Hib combination vaccine (acellular pertussis, diphtheria, tetanus, and Hib), enterovirus 71 inactivated vaccine (EV71), group A and C meningococcal polysaccharide conjugate vaccine (MPV-AC), monovalent oral rotavirus attenuated live vaccine (Rotv-1), pentavalent reassortant oral rotavirus attenuated live vaccine (Rotv-5), group A/C meningococcal–Hib combination conjugate vaccine (MPV-AC-Hib), 13-valent pneumococcal conjugate vaccine (PCV13), tetanus toxoid (TT), inactivated poliovirus vaccine (IPV), cholera vaccine (Chol), bivalent human papillomavirus vaccine (HPV2), tetravalent human papillomavirus vaccine (HPV4), nonavalent human papillomavirus vaccine (HPV9), herpes zoster vaccine (HZV), and others.

### 2.3. Substitutions with Non-NIP Vaccines 

Non-NIP vaccines can be used in place of their NIP counterparts, as follows, HepB, HepA, MMR, and JE-I can replace the corresponding NIP vaccines (HepB, HepA, MMR, and live-attenuated Japanese encephalitis vaccine (JEV-L), respectively). IPV and DTaP-IPV/Hib can replace the oral poliovirus vaccine (OPV).

DTaP (non-NIP), DTaP-Hib, and DTaP-IPV/Hib can substitute for NIP DTaP. MPV-AC, MPV-ACYW135, and MenAC-Hib can replace the group A meningococcal polysaccharide vaccine (MPV-A) and the group A/C meningococcal polysaccharide vaccine (MPV-AC).

### 2.4. Data Analysis

After data cleaning, descriptive epidemiological methods were applied to calculate (a) the total number of non-NIP vaccine doses administered, (b) the average number of non-NIP doses per 10,000 population, and (c) the substitution rate for each vaccine. Substitution is analyzed at the person. The substitution rate was defined as follows: substitution rate (%) = [doses of substitutive non-NIP vaccine] ÷ [doses of substitutive non-NIP vaccine + doses of corresponding NIP vaccine] × 100%. Data were processed and analyzed with SPSS 22.0 and Microsoft Excel 2010. Categorical variables were compared by using the χ^2^ test (Cochran–Armitage trend tests). Multistage regression analysis using Joinpoint software (v5.4.0) was performed to explore the changing trends with the average annual percentage change (AAPC). A two-sided *p* < 0.05 was considered to be statistically significant.

## 3. Results

### 3.1. Overall Utilization of Non-National Immunization Program (Non-NIP) Vaccines

From 2011 to 2024, a cumulative total of 91,009,259 doses of non-NIP vaccines were administered in Hubei Province, yielding an annual average of 6,500,661 doses per year. The highest annual number of doses (13,971,544) was recorded in 2023, whereas the lowest (3,784,730) occurred in 2011. On a per capita basis, the overall utilization rate was 1113.14 doses per 10,000 population. This rate rose from 657.07 doses per 10,000 population in 2011 to a peak of 2393.21 doses per 10,000 population in 2023—an increase of 264.22% (trend χ^2^ = 138.62, *p* < 0.05) (AAPC = 10.92%, *p* < 0.05)—before declining to 1755.03 doses per 10,000 population in 2024.

A total of 35 types of non-NIP vaccines were administered cumulatively, expanding from 23 in 2011 to 28 in 2024, peaking at 30 types in 2019. Non-NIP vaccines accounted for an overall average of 37.98% of all vaccine doses administered during the study period. This proportion grew from 25.52% in 2011 to 64.90% in 2024, representing a 154.33% increase (trend χ^2^ = 89.47, *p* < 0.05) (AAPC = 8.74%, *p* < 0.05). See Table 1.

### 3.2. Administration of Different Non-NIP Vaccines

From 2011 to 2024, the top 10 most frequently administered non-NIP vaccines in Hubei Province were as follows, in descending order: InfV 14,774,769 doses, RabV 10,836,334 doses, Hib 9,228,996 doses, VarV 8,167,184 doses, HepB 6,063,094 doses, EV71 5,614,044 doses, ORV1 3,595,019 doses, PPV23 3,470,414 doses, HPV2 2,822,454 doses, and IPV 2,601,357 doses.

Between 2011 and 2023, the administration of RabV, InfV, HepB, EV71, VarV, PPV23, and HPV2 showed upward trends. RabV exhibited the largest increase (2,060,949.65%; trend χ^2^ = 985.33, *p* < 0.05), followed by InfV (504.89%; trend χ^2^ = 204.71, *p* < 0.05) and HepB (1685.33%; trend χ^2^ = 312.45, *p* < 0.05). Conversely, doses of IPV, ORV1, and Hib declined by 94.62% (trend χ^2^ = 78.92, *p* < 0.05), 50.35% (trend χ^2^ = 45.61, *p* < 0.05), and 83.68% (trend χ^2^ = 66.84, *p* < 0.05), respectively.

**Figure 1:**
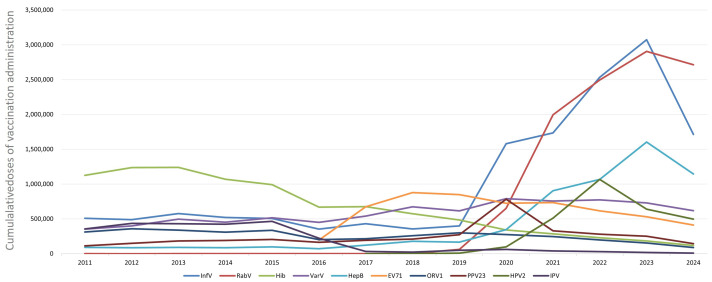
Trends in top 10 non-NIP vaccine administration in Hubei Province, central China, 2011–2024.

From 2023 to 2024, all ten of the above non-NIP vaccines experienced a decrease in the number of doses administered (Figure 1). Among the non-NIP vaccines, specific dose changes from 2023 to 2024 are as follows: IPV decreased from 19,170 doses in 2023 to 9675 doses in 2024, with a decline rate of 49.53% (χ^2^ = 485.22, *p* < 0.05); InfV dropped from 3,072,433 doses in 2023 to 1,714,084 doses in 2024, showing a 44.21% (χ^2^ = 621.34, *p* < 0.05) decline; ORV1 fell from 155,219 doses in 2023 to 87,698 doses in 2024, registering a 43.50% (χ^2^ = 298.45, *p* < 0.05) decline; PPV23 decreased from 250,490 doses in 2023 to 144,374 doses in 2024, with a 42.36% (χ^2^ = 275.11, *p* < 0.05) decline; Hib dropped from 183,902 doses in 2023 to 116,935 doses in 2024, showing a 36.41% (χ^2^ = 189.33, *p* < 0.05) decline; HepB fell from 1,604,240 doses in 2023 to 1,144,895 doses in 2024, with a 28.63% (χ^2^ = 162.78, *p* < 0.05) decline; HPV2 decreased from 640,772 doses in 2023 to 496,319 doses in 2024, registering a 22.54% (χ^2^ = 98.45, *p* < 0.05) decline; EV71 dropped from 531,595 doses in 2023 to 412,083 doses in 2024, showing a 22.48% (χ^2^ = 87.62, *p* < 0.05) decline; VarV fell from 729,657 doses in 2023 to 618,815 doses in 2024, with a 15.19% (χ^2^ = 56.33, *p* < 0.05) decline; and RabV decreased from 2,906,080 doses in 2023 to 2,713,662 doses in 2024, registering a 6.62% (χ^2^ = 32.14, *p* < 0.05) decline.

### 3.3. Non-NIP Vaccine Administration by Districts

From 2011 to 2024, the districts with the highest cumulative numbers of non-NIP vaccine doses administered in Hubei Province were Wuhan City (18,493,669 doses), Xiangyang City (8,790,521 doses), and Huanggang City (8,274,753 doses). The districts with the lowest totals were the Shennongjia Forest District (142,121 doses), Ezhou Autonomous Prefecture (1,170,955 doses), and Tianmen City (1,341,270 doses).

Among individual districts, the largest increases were observed in Wuhan City (480.58%; trend χ^2^ = 45.12, *p* < 0.05), followed by the Shennongjia Forest District (297.00%; trend χ^2^ = 38.91, *p* < 0.05) and Yichang City (227.69%; trend χ^2^ = 33.67, *p* < 0.05). The smallest increases occurred in Tianmen (86.36%; trend χ^2^ = 12.45, *p* < 0.05), Huanggang City (116.37%; trend χ^2^ = 15.78, *p* < 0.05), and Xiaogan City (118.38%; trend χ^2^ = 16.23, *p* < 0.05). See Table 2.

### 3.4. Administration of Non-NIP Vaccines in Children and Adults

From 2011 to 2024, a total of 44,376,048 doses of non-immunization program vaccines were administered to children in Hubei Province, with an average annual vaccination volume of 3,169,718 doses per year. The highest vaccination volume was in 2022 (3,817,855 doses), and the lowest was in 2011 (2,495,119 doses). The overall average per capita usage was 542.76 doses per 10,000 people, increasing from 433.18 doses per 10,000 people in 2011 to 653.29 doses per 10,000 people in 2022 (the highest usage), with an increase of 50.81% (trend χ^2^ = 28.45, *p* < 0.05). The types of vaccines administered increased from 13 in 2011 to 16 in 2024, with the highest number of types being in 2018 (17 types), as shown in Table 3.

From 2011 to 2024, a total of 187,746 doses of non-immunization program vaccines were administered to adults in Hubei Province, with an average annual vaccination volume of 13,410 doses per year. The highest vaccination volume was in 2023 (51,440 doses), and the lowest was in 2019 (20 doses). The overall average per capita usage was 2.29 doses per 10,000 people, increasing from 1.28 doses per 10,000 people in 2011 to 8.81 doses per 10,000 people in 2023 (the highest usage), with an increase of 588.28% (trend χ^2^ = 41.88, *p* < 0.05). The types of vaccines administered decreased from four in 2011 to two in 2024, with the highest number of types being in 2012, 2013, and 2016 (four types), as shown in Table 3.

From 2011 to 2024, a total of 44,703,949 doses of non-immunization program vaccines were administered to both children and adults in Hubei Province, with an average annual vaccination volume of 3,193,139 doses per year. The highest vaccination volume was in 2023 (10,250,423 doses), and the lowest was in 2016 (731,389 doses). The overall average per capita usage was 546.78 doses per 10,000 people, increasing from 156.97 doses per 10,000 people in 2011 to 1755.80 doses per 10,000 people in 2023 (the highest usage), with an increase of 1118.56% (trend χ^2^ = 132.67, *p* < 0.05). The types of vaccines administered increased from 7 in 2011 to 10 in 2024, with the highest number of types being in 2018 (11 types), as shown in Table 3.

### 3.5. Administration of Substitutive Non-NIP Vaccines

**Figure 2:**
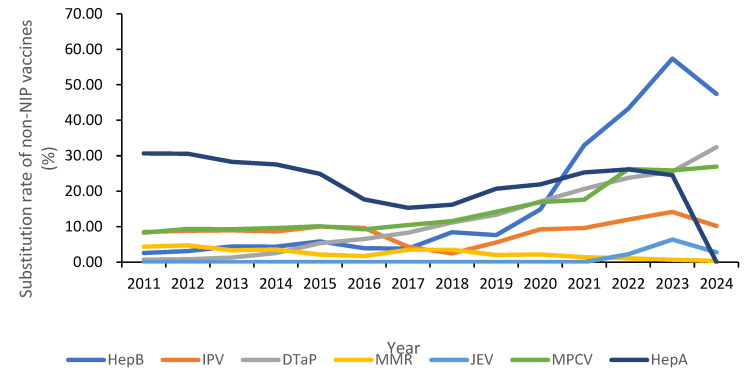
Substitution trends for substitutive rate of non-NIP vaccines, Hubei Province, central China, 2011–2023.

From 2011 to 2024, the substitutive non-NIP vaccines used in Hubei Province included HepB, polio, DTaP-containing, MMR, JE, meningococcal, and HepA, with a cumulative total of 16,618,755 doses administered. Annual doses rose from 651,004 in 2011 to 2,175,995 in 2024. The overall substitution rate increased from 5.57% in 2011 to 28.03% in 2024 (trend χ^2^ = 105.33, *p* < 0.05). HepB: substitution rate grew from 2.60% in 2011 to 47.42% in 2024 (increase: 1721.29%; trend χ^2^ = 88.76, *p* < 0.05). Polio vaccine: substitution rate rose from 8.50% in 2011 to 10.19% in 2024 (increase: 19.92%; trend χ^2^ = 12.45, *p* <0.05). DTaP-containing vaccines: substitution rate climbed from 0.73% in 2011 to 32.40% in 2024 (increase: 4345.85%; trend χ^2^ = 142.89, *p* < 0.01). JE vaccine: substitution rate increased from 0.01% in 2018 to 7.76% in 2023 (increase: 63,600.00%; trend χ^2^ = 68.91, *p* <0.05). Meningococcal vaccines: substitution rate rose from 8.32% in 2011 to 26.94% in 2024 (increase: 223.77%; trend χ^2^ = 45.67, *p* < 0.05). MMR vaccine: substitution rate declined from 4.36% in 2011 to 0.37% in 2023 (decrease: 91.58%; trend χ^2^ = 25.34, *p* < 0.05). HepA vaccine: substitution rate dropped from 30.65% in 2011 to 0.00% in 2024 (trend χ^2^ = 38.92, *p* < 0.05). See Table 4 and Figure 2.

From 2011 to 2023, a total of 14,442,760 doses of substitutive non-NIP vaccines were administered, with annual doses increasing from 651,004 in 2011 to 2,371,146 in 2023. The total substitution rate of alternative non-NIP vaccines rose from 5.57% (2011) to 24.74% (2023) (trend χ^2^ = 15.11, *p* < 0.05). Specific trends: HepB increased from 2.60% to 57.40% (increase: 2107.69%, trend χ^2^ = 25.58, *p* < 0.05); Polio vaccine increased from 8.50% to 14.15% (increase: 66.47%, trend χ^2^ = 6.33, *p* < 0.05); DTaP-containing vaccines increased from 0.73% to 25.54% (increase: 3398.63%, trend χ^2^ = 17.46, *p* < 0.05); JE vaccine increased from 0.01% (2018) to 6.37% (2023) (increase: 63600.00%, trend χ^2^ = 58.26, *p* < 0.05); Men increased from 8.32% to 25.86% (increase: 210.82%, trend χ^2^ = 22.66, *p* < 0.05); MMR decreased from 4.36% to 0.68% (decrease: 84.40%, trend χ^2^ = 19.59, *p* < 0.05); HepA vaccine decreased from 30.65% to 24.52% (decrease: 19.99%, trend χ^2^ = 3.25, *p* < 0.05).

From 2023 to 2024, cumulative doses of alternative non-NIP vaccines in Hubei Province reached 4,547,141, with annual doses decreasing from 2,371,146 (2023) to 2,175,995 (2024). The total substitution rate increased from 24.74% (2023) to 28.03% (2024). Specific trends: HepB decreased from 57.40% to 47.42% (decrease: 17.39%, trend χ^2^ = 15.67, *p* < 0.05); Polio vaccine decreased from 14.15% to 10.19% (decrease: 28.03%, trend χ^2^ = 8.45, *p* < 0.05); DTaP-containing vaccines increased from 25.54% to 32.40% (increase: 26.87%, trend χ^2^ = 12.34, *p* < 0.05); JE vaccine decreased from 7.76% to 2.77% (decrease: 64.29%, trend χ^2^ = 20.12, *p* < 0.05); Men increased from 25.86% to 26.94% (increase: 4.19%, trend χ^2^ = 1.23, *p* < 0.05); MMR decreased from 0.68% to 0.37% (decreased: 45.77%, trend χ^2^ = 5.67, *p* < 0.05); HepA decreased from 24.52% to 0.00% (trend χ^2^ = 50.45, *p* < 0.05).

## 4. Discussion

China’s Immunization Program has implemented the Expanded Program on Immunization (EPI) since 1978. From “4 vaccines preventing 6 diseases” in 1978 to “14 vaccines preventing 15 diseases” in 2024 [8,9], the types of vaccines included in the immunization program have been constantly enriched, and the scope of disease prevention has gradually expanded. According to the Hubei Province Non-Immunization Program Vaccine Vaccination Plan issued by the Hubei Provincial Health Commission, there are currently 44 types of non-immunization program vaccines in 23 categories available in Hubei Province, which can prevent 27 common infectious diseases. Some of these vaccines are beyond the coverage of immunization program vaccines and serve as an effective supplement to them, effectively reducing the occurrence and prevalence of corresponding infectious diseases [10,11,12,13,14,15].

From 2011 to 2023, both the absolute number and per capita usage of non-NIP vaccines increased significantly. The proportion of non-NIP vaccines among total doses rose from 25.52% to 65.95%, indicating a growing reliance on voluntary, self-paid vaccines. This pattern aligns with global trends in middle-income countries, where rising disposable income, increased health awareness, and expanded vaccine markets have driven demand for optional vaccines [16]. Similar transitions have been observed in Latin America, the Caribbean, and Malaysia [17,18], where private vaccine markets have expanded due to public demand for broader protection beyond national programs’ childhood vaccination coverage in Europe: the impact of different public health policies [15]. As observed in other upper-middle-income countries, private vaccine uptake tends to rise until saturation or policy integration occurs, followed by a plateau or decline. However, the sharp decline in non-NIP vaccine use in 2024—particularly for the influenza, IPV, and rotavirus vaccines—signals a potential inflection point. This downturn may be attributed to several interrelated factors such as increased vaccine hesitancy, declining birth rate, and changed income [19].

The vaccination volume of InfV increased rapidly from 2019 to 2023, which may be attributed to the country’s emphasis on respiratory infectious diseases by increasing publicity efforts [20] and the successive launch of new technologies such as quadrivalent subunit influenza vaccines and nasal spray attenuated live influenza vaccines [21,22,23,24]. The vaccination volume of RabV also increased rapidly after 2019, which is likely closely related to the publicity and education on the hazards of rabies through new media, the increase in the number of rabies vaccine manufacturers, technological advancements, and improved production capacity [25]. The surge in the use of rabies vaccines in recent years may be related to the improvement in rabies vaccine production capacity, the widespread establishment of rabies, the further intensification of publicity efforts, and the establishment and improvement of the immunization information system. It is possible that the data on rabies vaccine administration before 2020 was not fully incorporated into the Hubei Provincial Immunization Program Information System for statistical purposes. In 2023, the National Administration of Disease Prevention and Control issued the new Work Standards for Rabies Exposure Prophylaxis and Treatment [26].

However, the vaccination volume of Hib dropped from over one million doses before 2014 to more than 100,000 doses in 2024. The main reason is probably that in recent years, combined vaccines such as triple, quadruple, and pentavalent vaccines have included the Hib component [27,28,29,30], so parents and medical staff at vaccination sites no longer need to arrange separate Hib vaccinations. Since the bivalent human papillomavirus vaccine entered the market in 2017, its vaccination volume showed an upward trend until 2022 and a downward trend after 2022. This may be related to the significant increase in the import and batch release of the quadrivalent human papillomavirus vaccine and nonavalent human papillomavirus vaccine after 2022, which squeezed the market share of the bivalent vaccine [31].

The vaccination volume of IPV has been low since 2017, mainly due to the adjustment of the national immunization schedule for polio vaccines in recent years, which included IPV in the National Immunization Program [32,33]. From 2023 to 2024, the vaccination volume of most non-immunization program vaccines in Hubei Province showed a significant decline, mainly due to the following reasons. Firstly, having been affected by the mass vaccination of COVID-19 vaccines, the public has doubts about the effectiveness and safety of vaccines, leading to vaccination hesitation. In addition, negative information about vaccines has occasionally appeared online in recent years [34,35]. Secondly, the number of newborns has shown a downward trend in recent years [36]. Thirdly, since May 2023, the medical insurance department of Hubei Province has clearly stipulated that the balance of medical insurance cannot be used to pay for vaccine fees, resulting in the need to settle vaccine fees in cash only [37].

The results of this study show that there are significant differences in the growth of the number of non-immunization program vaccine doses administered across different regions in Hubei Province. For example, the growth rate in Wuhan and Yichang exceeds or is close to 300%, while that in Huanggang and Tianmen is less than 180%. The main reasons for the regional differences are probably as follows. Economic factors: The household income of the public is uneven, and the amount of money that can be allocated for vaccination is limited. Economically developed regions such as Wuhan and Yichang regularly allocate financial funds for free vaccination of key populations against influenza, pneumonia, shingles, etc. [20,38]. Subjective cognitive factors: Individuals with a high level of awareness and trust in vaccines have a stronger willingness to be vaccinated [39]. This group usually obtains information about vaccines through news media, popular science publicity, or recommendations from medical staff, and chooses the vaccines they need to be inoculated with. Media public opinion factors: Objective and rational positive reports can enhance the public’s understanding and awareness of vaccines, effectively promoting vaccination work [40]; on the contrary, repeated exposure and media hyping of negative incidents may trigger public opinion crises, leading to vaccine hesitation. Examples include the “Changchun Changsheng rabies vaccine incident” in 2018 and the “public opinion on Sinovac vaccines causing lung nodules” in 2022, which have affected vaccination. The results of this study indicate that the overall replacement rate of non-immunization program vaccines shows a slow annual growth trend. However, the replacement rate of some vaccines decreased significantly in 2024, mainly including the HepB Vaccine, Polio Vaccine, MMR Vaccine, Japanese Encephalitis Vaccine, and HepA Vaccine. The main reason is that the National Immunization Program vaccine schedule in Hubei Province has been updated year by year, incorporating non-immunization program vaccines such as the polio vaccine and hepatitis A vaccine into the immunization schedule. In addition, the regular vaccination protocols for vaccines such as the hepatitis B vaccine have been further optimized, reducing the need for non-immunization program vaccines. For another part of alternative non-immunization program vaccines, such as those containing DTaP (diphtheria, tetanus, and acellular pertussis) components, their replacement rate has increased year by year. The main social demand for non-immunization program vaccines tends to be multi-valent and multi-combination alternative vaccines [41], such as DTaP-IPV-Hib and MPV-ACYW135. This might be because these vaccines can reduce the number of vaccination doses for infants and lower transportation and time costs.

Given the sharp drop in non-National Immunization Program (non-NIP) vaccine use, the following management recommendations are put forward. First, financing and inclusion: increase government funding to include non-NIP vaccines like HPV, MPV-AC, and InfV in the National Immunization Program; when procuring National Immunization Program Vaccines, consider alternative options and cut unnecessary procurement to save costs [42]. Conduct in-depth cost-effectiveness research on vaccinated vs. unvaccinated populations to build a database, supporting efforts to cover vaccine costs under medical insurance and reduce public financial burden. Second, capacity and service quality: provide training for primary-level vaccination staff and evaluate facility hardware to boost their recognition of non-NIP vaccines, enhance service quality and accessibility, and standardize non-NIP vaccine transportation, storage, and administration to ensure supply safety, timeliness, and sufficiency, and prevent adverse events. Third, speed up the construction of adult vaccination clinics to meet adult demand for non-NIP vaccines. Use mass media to strengthen publicity for parents, improving their vaccine awareness and compliance. Additionally, in the later stage, strengthen investigations into the causes of the sharp drop in non-NIP vaccine use (e.g., public vaccine hesitancy, declining newborn numbers, medical insurance policy adjustments) to provide targeted support for optimizing subsequent management.

This study has several limitations. First, the analysis is limited to Hubei Province, and the findings may not be fully generalizable to other regions of China with different socioeconomic conditions or immunization policies. Second, data were extracted from the immunization information system and may not capture vaccines administered in private clinics outside the system. Third, the study did not assess the impact of non-NIP vaccine utilization on disease incidence, which would provide further evidence of their public health value. Future research should address these gaps by conducting multicenter studies, collecting individual-level data, and evaluating disease outcomes.

## 5. Conclusions

The utilization of non-NIP vaccines in Hubei Province has undergone significant changes over the past 14 years, characterized by long-term growth, a sharp 2024 decline, and evolving substitution patterns. Strategic investments in public financing, health communication, and service delivery are essential to sustain vaccine coverage and equity in China.

## Figures and Tables

**Table 1:** Utilization of non-National Immunization Program (Non-NIP) vaccines in Hubei Province, central China, 2011–2024.

Year	Number of Vaccine Types	Population (10,000 Persons)	Non-NIP Vaccine Doses	Proportion of Non-NIP Vaccines (%)	Per Capita Utilization of Non-NIP Vaccines (Doses/10,000 Population)
2011	23	5760	3,784,730	25.52	657.07
2012	25	5781	4,141,160	24.38	716.34
2013	25	5798	4,314,150	25.11	744.08
2014	24	5816	4,026,918	24.31	692.39
2015	24	5850	4,173,382	24.92	713.40
2016	27	5885	3,125,483	20.91	531.09
2017	28	5904	4,029,452	23.96	682.50
2018	30	5917	4,387,819	26.63	741.56
2019	30	5927	4,774,901	29.88	805.62
2020	28	5775	7,612,584	43.41	1318.20
2021	28	5830	9,934,268	53.63	1703.99
2022	27	5844	12,494,007	62.18	2137.92
2023	27	5838	13,971,544	65.95	2393.21
2024	28	5834	10,238,861	64.90	1755.03
Average	35	5840	6,500,661	37.98	1113.14

**Table 2:** Non-NIP vaccine administration by district in Hubei Province, central China, 2011–2024.

District	Cumulative Doses (2011–2024)	Doses in 2011	Doses in 2024	Increase (%)
Wuhan City	18,493,669	500,762	2,907,308	480.58
Huangshi City	3,276,900	187,534	346,204	84.61
Shiyan City	5,231,190	254,528	621,176	144.05
Yichang City	4,477,825	198,243	649,621	227.69
Xiangyang City	8,790,521	449,574	979,797	117.94
Ezhou City	1,170,955	59,408	154,802	160.57
Jingmen City	3,234,638	174,668	366,531	109.84
Xiaogan City	5,489,735	291,669	636,960	118.38
Jingzhou City	7,188,469	410,665	777,796	89.40
Huanggang City	8,274,753	432,590	935,984	116.37
Xianning City	3,661,528	206,442	418,850	102.89
Suizhou City	2,359,823	114,138	303,913	166.27
Enshi Autonomous Prefecture	4,355,147	220,854	538,740	143.93
Xiantao	1,821,521	114,883	268,354	133.59
Qianjiang City	1,460,333	79,327	157,298	98.29
Tianmen Autonomous Prefecture	1,341,270	85,249	158,869	86.36
Shennongjia Forest District	142,121	4196	16,658	297.00
Total	80,770,398	3,784,730	10,238,861	170.53

**Table 3:** Administration of non-NIP vaccines in children and adults, Hubei Province, central China, 2011–2023.

Types of Vaccines	2011	2012	2013	2014	2015	2016	2017	2018	2019	2020	2021	2022	2023	2024
**Vaccines only for children**														
Subtotal of vaccine doses (only for children)	2,495,119	2,893,522	2,950,745	2,764,276	2,954,322	2,266,925	3,050,409	3,416,424	3,621,217	3,747,499	3,740,072	3,817,855	3,663,276	2,994,387
Subtotal of vaccine types (only for children)	13	14	14	14	14	16	16	17	17	16	15	15	15	16
**Vaccines only for adults**														
Subtotal of vaccine doses (only for adults)	7370	5366	11,104	9077	9819	3459	4220	219	21	1598	11,894	35,474	51,440	36,693
Subtotal of vaccine types (only for adults)	3	4	4	3	3	4	3	2	3	2	2	2	2	2
**Vaccines for both children and adults**														
Subtotal of vaccine doses (for both adults and children)	904,162	956,514	1,102,166	1,036,501	1,034,821	731,389	864,919	889,349	1,100,217	3,813,551	6,171,569	8,640,642	10,250,423	7,207,726
Subtotal of vaccine types (for both adults and children)	7	7	7	7	7	7	9	11	10	10	10	10	10	10
Total of vaccine doses	3,784,730	4,141,160	4,314,150	4,026,918	4,173,382	3,125,483	4,029,452	4,387,819	4,774,901	7,612,584	9,934,268	12,494,007	13,971,544	10,238,806
Total of vaccine types	24	26	26	25	24	28	27	30	31	28	28	27	27	28

**Table 4:** Administration of substitutive non-NIP vaccines, Hubei Province, central China, 2011–2024.

Year	Doses of Substitutive Non-EPI Vaccines	Doses of EPI Vaccines Replaced	Substitution Rate (%)
2011	651,004	11,046,036	5.57%
2012	794,998	12,844,473	5.83%
2013	823,368	12,864,922	6.02%
2014	844,782	12,535,131	6.31%
2015	946,738	12,574,205	7.00%
2016	768,901	11,819,310	6.11%
2017	780,419	12,786,676	5.75%
2018	884,305	12,089,572	6.82%
2019	1,007,996	11,203,367	8.25%
2020	1,237,888	9,922,820	11.09%
2021	1,513,621	8,590,187	14.98%
2022	1,817,594	7,600,859	19.30%
2023	2,371,146	7,213,081	24.74%
2024	2,175,995	5,587,471	28.03%

## Data Availability

The datasets analyzed during the current study are not publicly available due to containing sensitive participant information that could compromise privacy or restrictions imposed by the funding agreement with but are available from the corresponding author upon reasonable request.
